# Prevalence and correlates of hyperuricemia in the middle-aged and older adults in China

**DOI:** 10.1038/s41598-018-22570-9

**Published:** 2018-03-12

**Authors:** Peige Song, He Wang, Wei Xia, Xinlei Chang, Manli Wang, Lin An

**Affiliations:** 1 Department of Maternal and Child Health, School of Public Health, Peking University, Beijing, China; 2 Centre for Global Health Research, University of Edinburgh, Edinburgh, UK; 3 The First Affiliated Hospital of Zhengzhou University, Zhengzhou, China; 4 School of Nursing, University of Hong Kong, Hong Kong, China

## Abstract

Hyperuricemia, the physiological prerequisite for gout, is linked to the presence and severity of multiple comorbidities that affect longevity and well-being. By using the baseline data from the China Health and Retirement Longitudinal Study, a nationally representative survey, the prevalence of hyperuricemia in general middle-aged and older Chinese was estimated. The potential effects of health behaviours and comorbidities on hyperuricemia were also explored. In 2010, the prevalence of hyperuricemia among middle-aged and older Chinese was 6.4%. Hyperuricemia was more prevalent in males than in females (7.9% vs. 4.9%). The risk of hyperuricemia increased with advanced age in both sexes. In males, current drinking, obesity and dyslipidemia were positively associated with hyperuricemia, whereas singles males and males living in North China were with lower odds of having hyperuricemia. For females, being single, at a higher economic level, living in the Southwest China, smoking, obesity, diabetes, hypertension and dyslipidemia were all significant risk factors for hyperuricemia, but females living in North China and Northwest China were with a lower hyperuricemia prevalence than females in East China. Therefore, hyperuricemia in China was not as prevalent as in developed countries, its prevalence varied greatly according to demographic, socioeconomic, and geographic factors.

## Introduction

Uric acid is the end product of purine metabolism in human^1^. Hyperuricemia, defined as the presence of an elevated serum uric acid (SUA) concentration, can lead to urate deposit in joints, tendons, and other tissues^2,3^. As the physiological prerequisite for gout^4^, hyperuricemia seems to be also linked to the presence and severity of multiple comorbidities that affect longevity and well-being, such as hypertension, cardiovascular disease, diabetes and metabolic syndrome^2,5–9^, placing a considerable public health burden on the society.

Hyperuricemia is highly prevalent in developed countries^10,11^. According to the results from the National Health and Nutrition Examination Survey (NHANES), around 21% (or 43 million) American adults aged 20 years and above were with hyperuricemia^11^. Compared with developed countries, the prevalence of hyperuricemia tends to be lower in developing countries^4^. For example, the prevalence of hyperuricemia is 10.6% in Thailand and 8.4% in Saudi Arabia^12,13^. For the largest developing country - China, where rapid ageing and urbanization are underway^14–16^, increased inactivity at work and leisure has occurred, and the prevalence of noncommunicable diseases (NCDs), obesity and the morbidity with NCDs have risen in the past decades^15,17,18^. Under such circumstances, a relatively larger burden of hyperuricemia is expected compared with other developing countries. To date, various epidemiological studies have been conducted to reveal the prevalence of hyperuricemia across China, however, these estimates were contingent upon different characteristics of individual studies, such as the age structure of the study sample, geographic regions of the study sites, etc.^19,20^. Therefore, well-designed large-scale investigations are called for to give a better estimation of the hyperuricemia prevalence in China.

The first large-scale estimation of hyperuricemia prevalence was based on the China National Survey of Chronic Kidney Disease, however, that study was not designed specifically to portray a complete picture of the hyperuricemia prevalence in China, for example, no age-stratified or region-specific prevalence estimates of hyperuricemia were demonstrated^21^. More notably, the China National Survey of Chronic Kidney Disease was conducted in only 13 selected provinces, the distinct regional and economic diversity within the whole nation cannot be well-addressed^22^. Therefore, a nationally representative sample of Chinese general population is urgently needed to provide better estimates and to validate the findings in previous studies.

In this study, we estimated the prevalence of hyperuricemia by using the China Health and Retirement Longitudinal Study (CHARLS), a nationally representative sample of Chinese middle-aged and older adults. The objectives of this study are: 1) to present the prevalence of hyperuricemia in general Chinese people aged 45 years and above, according to the demographic characteristic, socioeconomic status, and geographic location; 2) to identify potential associated factors and comorbidities of hyperuricemia in the middle-aged and older Chinese.

## Results

The CHARLS 2011 included 17,708 individuals from 10,257 households, of whom blood samples were collected from 11,847 participants. In the current study, a total of 9,557 subjects aged 45 years and above and with complete information on demographic characteristics, socioeconomic status, geographic location, and SUA concentration, were included for the stratification analysis of hyperuricemia prevalence. The patterns of age, sex, education, economic level distribution were similar between the included and non-included subjects. The distribution of marital status and geographic region in the included subjects was different from that in the non-included subjects, but similar to that in the whole sample (Supplementary Table S1).

### Participant Characteristics

The background characteristics of the 9,557 subjects are shown in Table 1. 52.4% of the subjects were females, and more than one third (36.1%) received middle school education and above. The majority (87.3%) of the study participants were married or cohabiting, and more than half (53.2%) were living in rural areas. East China and South Central China together owed more than half (54.1%) of the study participants. The patterns of age, setting and geographic region were similar between males and females. For education distribution, almost half (45.2%) of the male participants received middle school education and above, whereas more than one-third (37.7%) of the female participants were illiterate. In addition, a larger proportion (16.4%) of the female participants were single compared with males (8.9%).

**Table 1:** General characteristics of the included subjects in the CHARLS 2011 survey^*^.

Characteristic	Male (n = 4,546)	Female (n = 5,011)	Overall (n = 9,557)
**Age group**			
45–49 years	786 (20.9)	1,079 (22.7)	1,865 (21.9)
50–59 years	1,583 (34.9)	1,815 (34.1)	3,398 (34.5)
60–69 years	1,390 (26.0)	1,369 (25.0)	2,759 (25.5)
≥ 70 years	787 (18.1)	748 (18.2)	1,535 (18.2)
**Education**			
Illiterate	612 (11.5)	2,110 (37.7)	2,722 (24.9)
Literate	868 (17.8)	893 (16.5)	1,761 (17.1)
Primary education	1,227 (25.5)	887 (18.4)	2,114 (21.9)
Middle school education and above	1,839 (45.2)	1,121 (27.4)	2,960 (36.1)
**Marital status**			
Married or cohabiting	4,151 (91.1)	4,322 (83.6)	8,473 (87.3)
Single	395 (8.9)	689 (16.4)	1,084 (12.7)
**Ln(PCE) by setting** ^**†**^			
*Rural*	*2*,*950 (53*.*6)*	*3*,*177(52*.*9)*	*6*,*127 (53*.*2)*
Bottom tertile	978 (17.7)	1,090 (18.4)	2,068 (18.1)
Middle tertile	999 (17.9)	1,068 (17.2)	2,067 (17.5)
Top tertile	973 (18.0)	1,019 (17.2)	1,992 (17.6)
*Urban*	*1*,*596 (46*.*4)*	*1*,*834 (47*.*1)*	*3*,*430 (46*.*8)*
Bottom tertile	574 (12.5)	682 (13.6)	1,256 (13.1)
Middle tertile	537 (14.5)	598 (14.5)	1,135 (14.5)
Top tertile	485 (19.4)	554 (19.0)	1,039 (19.2)
**Region**			
East China	1,281 (28.6)	1,480 (29.2)	2,761 (28.9)
North China	688 (12.8)	725 (12.8)	1,413 (12.8)
Northeast China	348 (8.9)	402 (9.0)	750 (9.0)
Northwest China	398 (8.4)	401 (7.8)	799 (8.1)
South Central China	989 (24.8)	1,141 (25.5)	2,130 (25.2)
Southwest China	842 (16.5)	862 (15.7)	1,704 (16.1)

^*^Values were the number of subjects (weighted proportion). PCE = per capita expenditure.

^†^The bottom tertile of Ln(PCE) refers to the poor, the middle tertile refers to the middle, and the top tertile refers to the rich.

### Prevalence of hyperuricemia

**Figure 1:**
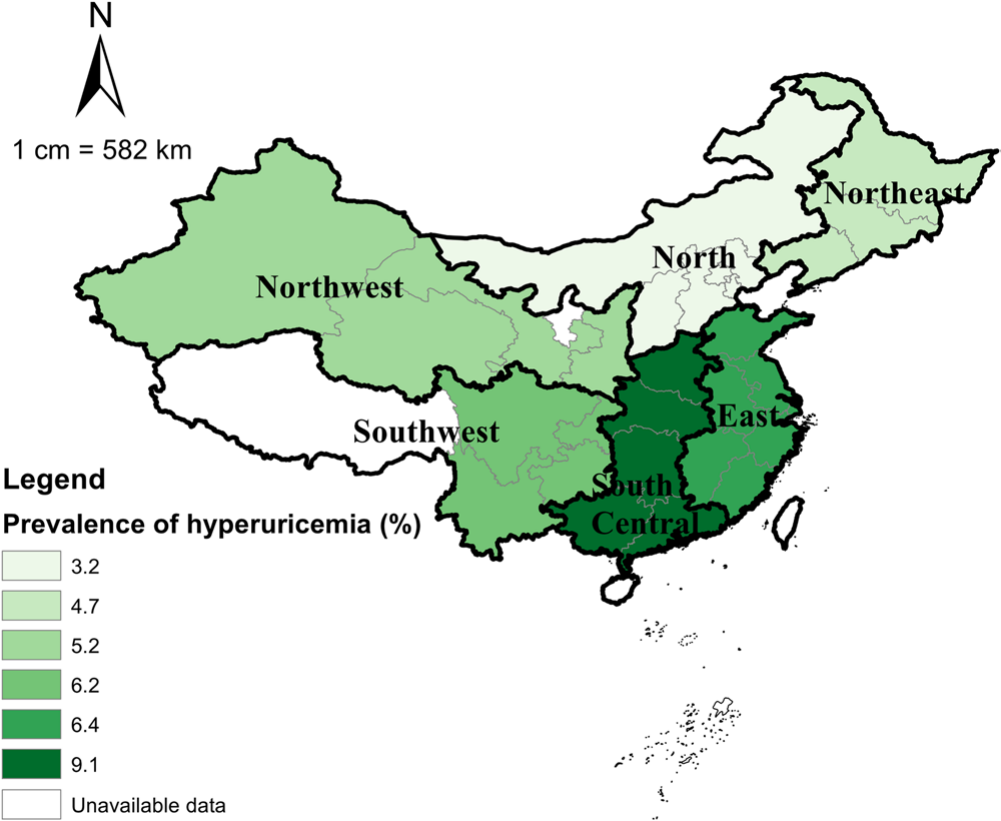
Prevalence of hyperuricemia in the CHARLS 2011 survey, by geographic region. The map was created using ArcMap (version 10.1, ESRI Inc. Redlands, CA, USA. https://www.esri.com/).

As demonstrated in Table 2, the mean SUA level was 4.57 mg/dl overall, with a mean level of 5.04 mg/dl in males and 4.12 mg/dl in females. The age-standardized level of SUA was 4.55 mg/dl overall. The overall prevalence of hyperuricemia was 6.4%, which translated to an estimated 28.4 million middle-aged and older adults with hyperuricemia in China in 2010. The age-standardized prevalence of hyperuricemia was 6.2%. Hyperuricemia was more common in males than in females, with the crude prevalence of 7.9% and 4.9% and the age-standardized prevalence of 7.8% and 4.8% respectively. The prevalence of hyperuricemia increased from 3.5% among participants aged 45–49 years to 10.3% among participants aged 70 years and older. The prevalence of hyperuricemia was greater in urban areas (8.0%) than in rural areas (5.0%). Participants that attained primary education were with the highest prevalence of hyperuricemia (8.1%) and illiterate participants were with the lowest (5.3%). In both urban and rural settings, the prevalence of hyperuricemia increased with the improvement of economic levels, ranging from 3.6% in the poor (the bottom tertile of Ln[PCE]) to 5.9% in the rich (the top tertile of Ln[PCE]) in rural areas, and from 7.0% in the urban poor to 8.8% in the urban rich. The prevalence of hyperuricemia differed slightly among those who were married or cohabiting (6.2%) and single participants (7.4%). Geographically, the prevalence of hyperuricemia was the highest among people living in South Central China (9.1%) and the lowest among those in North China (3.2%) (also see Fig. 1 and Supplementary Figure S1).

**Table 2:** Mean serum uric acid concentration and prevalence of hyperuricemia among the included subjects in the CHARLS 2011 survey^*^.

	Serum uric acid concentration, mean ± SD, mg/dl			Prevalence of hyperuricemia, % (95% CI)		
	Male	Female	Overall	Male	Female	Overall
**Crude**	5.04 ± 0.05	4.12 ± 0.04	4.57 ± 0.04	7.9 (5.8–10.0)	4.9 (3.9–5.9)	6.4 (5.2–7.6)
**Age-standardized**	5.03 ± 0.05	4.11 ± 0.04	4.55 ± 0.04	7.8 (5.8–9.8)	4.8 (3.8–5.7)	6.2 (5.1–7.4)
**Age group**						
45–49 years	4.86 ± 0.12	3.81 ± 0.05	4.30 ± 0.07	4.8 (3.1–6.4)	2.4 (1.2–3.5)	3.5 (2.5–4.5)
50–59 years	4.94 ± 0.07	4.07 ± 0.05	4.50 ± 0.05	7.0 (4.1–9.9)	3.8 (2.5–5.0)	5.4 (3.7–7.1)
60–69 years	5.03 ± 0.06	4.25 ± 0.08	4.64 ± 0.05	7.9 (6.0–9.8)	6.8 (4.7–8.9)	7.4 (5.9–8.9)
≥70 years	5.43 ± 0.22	4.39 ± 0.07	4.90 ± 0.13	13.3 (4.1–22.4)	7.6 (5.2–10.0)	10.3 (5.5–15.1)
**Education**						
Illiterate	4.87 ± 0.07	4.11 ± 0.04	4.28 ± 0.04	5.9 (3.8–8.1)	5.2 (3.9–6.5)	5.3 (4.2–6.5)
Literate	5.00 ± 0.08	4.08 ± 0.06	4.55 ± 0.06	6.8 (4.5–9.2)	5.1 (3.1–7.2)	6.0 (4.6–7.4)
Primary education	5.23 ± 0.17	4.17 ± 0.11	4.77 ± 0.14	11.2 (4.1–18.4)	4.1 (2.1–6.0)	8.1 (3.9–12.4)
Middle school education and above	4.99 ± 0.07	4.11 ± 0.04	4.65 ± 0.06	6.9 (5.1–8.8)	5.0 (3.2–6.7)	6.2 (4.7–7.7)
**Marital status**						
Married or cohabiting	5.06 ± 0.05	4.07 ± 0.04	4.57 ± 0.04	8.1 (5.9–10.4)	4.2 (3.2–5.3)	6.2 (4.9–7.5)
Single	4.82 ± 0.09	4.35 ± 0.07	4.51 ± 0.06	5.6 (3.2–7.9)	8.4 (5.8–11.0)	7.4 (5.5–9.4)
**Ln(PCE) by setting** ^**†**^						
*Rural*	*4*.*87 ± 0*.*05*	*3*.*96 ± 0*.*04*	*4*.*41 ± 0*.*04*	*6*.*1 (4*.*9–7*.*3)*	*3*.*9 (2*.*9–4*.*9)*	*5*.*0 (4*.*1–5*.*8)*
Bottom tertile	4.73 ± 0.07	3.90 ± 0.05	4.30 ± 0.05	4.9 (3.3–6.4)	2.4 (1.47–3.3)	3.6 (2.7–4.4)
Middle tertile	4.85 ± 0.06	4.00 ± 0.06	4.42 ± 0.05	6.3 (4.7–7.9)	4.7 (3.1–6.4)	5.5 (4.2–6.9)
Top tertile	5.02 ± 0.07	4.00 ± 0.05	4.51 ± 0.05	7.1 (4.9–9.3)	4.6 (2.9–6.4)	5.9 (4.4–7.3)
*Urban*	*5*.*23 ± 0*.*07*	*4*.*29 ± 0*.*05*	*4*.*74 ± 0*.*05*	*10*.*0 (6*.*0–14*.*0)*	*6*.*0 (4*.*3–7*.*8)*	*8*.*0 (5*.*8–10*.*1)*
Bottom tertile	5.04 ± 0.09	4.20 ± 0.07	4.59 ± 0.07	8.2 (4.9–11.4)	5.9 (3.2–8.6)	7.0 (4.5–9.4)
Middle tertile	5.26 ± 0.11	4.30 ± 0.08	4.77 ± 0.08	8.7 (4.0–13.3)	7.0 (4.6–9.4)	7.8 (4.9–10.7)
Top tertile	5.32 ± 0.10	4.35 ± 0.07	4.83 ± 0.06	12.2 (4.7–19.7)	5.5 (2.7–8.2)	8.8 (5.6–11.9)
**Region**						
East China	5.06 ± 0.08	4.22 ± 0.06	4.63 ± 0.06	7.5 (5.2–9.8)	5.3 (3.4–7.2)	6.4 (4.8–7.9)
North China	4.61 ± 0.09	3.79 ± 0.06	4.19 ± 0.06	3.5 (2.2–4.9)	2.8 (1.5–4.1)	3.2 (2.5–3.8)
Northeast China	4.90 ± 0.14	3.99 ± 0.12	4.43 ± 0.11	5.0 (1.9–8.0)	4.5 (1.6–7.5)	4.7 (2.3–7.2)
Northwest China	4.95 ± 0.17	3.88 ± 0.09	4.42 ± 0.13	8.0 (2.8–13.1)	2.4 (0.0–5.0)	5.2 (1.8–8.7)
South Central China	5.28 ± 0.12	4.26 ± 0.08	4.75 ± 0.10	12.7 (6.0–19.4)	5.7 (3.0–8.4)	9.1 (5.5–12.7)
Southwest China	5.07 ± 0.08	4.17 ± 0.08	4.62 ± 0.07	6.4 (4.9–7.9)	6.0 (4.8–7.3)	6.2 (5.3–7.2)

^*^Values were weighted. 95% CI = 95% confidence interval PCE = per capita expenditure.

^†^The bottom tertile of Ln(PCE) refers to the poor, the middle tertile refers to the middle, and the top tertile refers to the rich.

The Prevalence of SUA ≥ 7 mg/dl regardless of sex was 4.5% overall, with a prevalence of 7.9% among males and 1.3% among females. The prevalence of SUA ≥ 6 mg/dl regardless of sex was 12.5% overall, with a prevalence of 20.5% in males and 4.9% in females (Supplementary Table S2).

### Associated factors and comorbidities of hyperuricemia

In the univariable logistic regression analyses (Table 3), obesity and dyslipidemia were positively, and residing in the North China region was negatively, associated with the presence of hyperuricemia in males. In females, advanced age, being single, at a higher economic level, former smoking, former drinking, obesity, diabetes, hypertension and dyslipidemia were all positive correlates of hyperuricemia. After adjusting for demographic characteristics, socioeconomic status, geographic locations, health behaviours and comorbidities (Table 3), it turned out that older age, current drinking, obesity and dyslipidemia were positively associated with hyperuricemia in males, whereas singles males and males living in North China were with lower odds of having hyperuricemia. For females, being older, being single, at a higher economic level, living in the Southwest China, smoking (former and current smoking), obesity, diabetes, hypertension and dyslipidemia were all with higher risks of hyperuricemia, in the meanwhile, the prevalence of hyperuricemia was much lower in females living in North China and Northwest China than those in East China.

**Table 3:** Odds ratios for hyperuricemia in middle-aged and older Chinese, as determined by univariable and multivariable logistic regression analyses^*^.

	Male		Female	
	Model 1	Model 2	Model 1	Model 2
**Age group**				
45–49 years	1.00 (reference)	1.00 (reference)	1.00 (reference)	1.00 (reference)
50–59 years	1.08 (0.49–2.38)	1.27 (0.58–2.74)	2.17 (1.12–4.20)^‡^	1.80 (0.94–3.44)
60–69 years	1.56 (0.95–2.55)	1.83 (1.01–3.29)^‡^	3.99 (1.98–8.06)^‡^	3.21 (1.64–6.30)^‡^
≥70 years	2.91 (0.89–9.54)	3.11 (1.37–7.09)^‡^	4.92 (2.62–9.25)^‡^	3.24 (1.82–5.76)^‡^
**Education**				
Illiterate	1.00 (reference)	1.00 (reference)	1.00 (reference)	1.00 (reference)
Literate	1.24 (0.73–2.09)	1.26 (0.72–2.21)	0.89 (0.57–1.37)	0.94 (0.57–1.55)
Primary education	2.37 (0.94–5.94)	1.62 (0.87–3.00)	0.75 (0.44–1.29)	0.80 (0.47–1.39)
Middle school education and above	1.17 (0.66–2.07)	1.15 (0.60–2.20)	0.86 (0.51–1.44)	1.37 (0.77–2.42)
**Marital status**				
Married or cohabiting	1.00 (reference)	1.00 (reference)	1.00 (reference)	1.00 (reference)
Single	0.60 (0.33–1.10)	0.57 (0.32–0.99)^‡^	2.40 (1.54–3.74)^‡^	1.73 (1.04–2.88)^‡^
**Ln(PCE) by setting** ^**†**^				
*Rural*				
Bottom tertile	1.00 (reference)	1.00 (reference)	1.00 (reference)	1.00 (reference)
Middle tertile	1.18 (0.74–1.86)	1.22 (0.75–1.99)	2.66 (1.54–4.58)^‡^	2.94 (1.64–5.26)^‡^
Top tertile	1.24 (0.78–1.96)	1.36 (0.78–2.34)	2.21 (1.21–4.03)^‡^	2.62 (1.41–4.86)^‡^
*Urban*				
Bottom tertile	1.48 (0.80–2.71)	1.16 (0.61–2.22)	2.97 (1.56–5.63)^‡^	2.34 (1.17–4.67)^‡^
Middle tertile	1.52 (0.67–3.47)	1.09 (0.48–2.48)	4.05 (2.05–7.98)^‡^	3.92 (1.83–8.40)^‡^
Top tertile	2.48 (0.94–6.54)	1.52 (0.74–3.10)	2.58 (1.13–5.90)^‡^	2.56 (1.08–6.03)^‡^
**Region**				
East China	1.00 (reference)	1.00 (reference)	1.00 (reference)	1.00 (reference)
North China	0.43 (0.25–0.76)^‡^	0.34 (0.19–0.63)^‡^	0.56 (0.27–1.15)	0.43 (0.22–0.85)^‡^
Northeast China	0.60 (0.27–1.34)	0.51 (0.22–1.19)	0.89 (0.31–2.56)	0.68 (0.23–2.00)
Northwest China	1.02 (0.43–2.41)	1.07 (0.39–2.93)	0.31 (0.09–1.03)	0.31 (0.11–0.88)^‡^
South Central China	1.96 (0.90–4.25)	1.56 (0.85–2.84)	1.13 (0.57–2.23)	1.50 (0.72–3.13)
Southwest China	0.82 (0.50–1.33)	0.94 (0.57–1.54)	1.29 (0.76–2.17)	1.98 (1.07–3.69)^‡^
**Smoking**				
Never smoker	1.00 (reference)	1.00 (reference)	1.00 (reference)	1.00 (reference)
Former smoker	0.59 (0.21–1.64)	0.69 (0.38–1.25)	3.17 (1.42–7.06)^‡^	3.17 (1.44–6.97)^‡^
Current smoker	0.45 (0.20–1.01)	0.66 (0.43–1.01)	1.54 (0.84–2.85)	1.97 (0.96–4.08)^‡^
**Drinking**				
Never drinker	1.00 (reference)	1.00 (reference)	1.00 (reference)	1.00 (reference)
Former drinker	1.01 (0.56–1.84)	1.03 (0.57–1.88)	3.10 (1.35–7.11)^‡^	2.19 (0.99–4.86)
Current drinker	1.53 (0.88–2.67)	1.70 (1.11–2.61)^‡^	1.09 (0.52–2.28)	1.06 (0.53–2.12)
**Obesity**				
Normal	1.00 (reference)	1.00 (reference)	1.00 (reference)	1.00 (reference)
Underweight	0.49 (0.22–1.06)	0.49 (0.23–1.03)	0.75 (0.33–1.71)	0.81 (0.36–1.83)
Overweight	1.24 (0.84–1.83)	1.04 (0.60–1.78)	1.11 (0.69–1.79)	0.96 (0.58–1.61)
Obese	4.81 (1.69–13.68)^‡^	2.63 (1.32–5.26)^‡^	2.78 (1.71–4.52)^‡^	2.64 (1.63–4.28)^‡^
**Diabetes**	0.99 (0.56–1.77)	0.73 (0.43–1.24)	2.62 (1.70–4.03)^‡^	1.87 (1.17–2.99)^‡^
**Hypertension**	1.27 (0.71–2.26)	1.13 (0.74–1.72)	2.72 (1.84–4.00)^‡^	1.77 (1.22–2.58)^‡^
**Dyslipidemia**	3.41 (1.89–6.14)^‡^	2.80 (1.86–4.23)^‡^	3.01 (2.19–4.13)^‡^	2.18 (1.55–3.08)^‡^

^*^Values are the weighted odds ratio (95% confidence interval). 95% CI = 95% confidence interval. PCE = per capita expenditure. Model 1 was a univariable logistic regression. Model 2 was a multivariable logistic regression, adjusted for all variables (education, marital status, Ln(PCE) by setting, region, smoking, drinking, obesity, central obesity, diabetes, hypertension and dyslipidemia). ^†^The bottom tertile of Ln(PCE) refers to the poor, the middle tertile refers to the middle, and the top tertile refers to the rich. ^‡^P < 0.05.

## Discussion

In this large population-based study involving a nationally representative sample of the middle-aged and older Chinese, the prevalence of hyperuricemia was estimated as 6.4%, corresponding to a total of 28.4 million affected Chinese people aged 45 years and above in 2010. We observed that the odds of hyperuricemia in males varied significantly with age group, marital status and geographic region; males who were currently drinking had a higher risk of having hyperuricemia, and males with obesity or dyslipidemia were more likely to have hyperuricemia. For females, the odds of hyperuricemia also varied significantly with age group, marital status and geographic region, notably; furthermore, the economic status played an important role in the development of hyperuricemia in females, with richer females at a higher risk of having hyperuricemia. Smoking brought a higher risk of hyperuricemia in females. Obesity, diabetes, hypertension and dyslipidemia were all additionally identified as significant comorbidities of hyperuricemia in females.

To our knowledge, our study provides the most comprehensive nationally representative estimates of the prevalence of hyperuricemia in middle-aged and older Chinese. The large geographic areas the CHARLS covered (28 out of the 31 provinces in Mainland China) granted us a good opportunity to assess the effects of demographic, socioeconomic and geographic features on the prevalence of hyperuricemia in a large developing country with substantial socioeconomic diversity. Furthermore, the reliability and credibility of our results can be largely assured by the standardized study protocol and data collection procedures, strict data collection training and quality control. Measurement bias of HUA can be substantially avoided in this study, benefiting from the fact that all the blood specimens were analysed in the same clinical laboratory according to a preset protocol.

In this study, our prevalence estimates were somewhat much lower than that from the latest meta-analysis of hyperuricemia prevalence in China that compiled data from all available individual studies in Chinese population from the year 2000 onwards, where an overall prevalence of hyperuricemia of 13.3% was reported^20^. The most likely reason is that the individual studies included in that meta-analysis were generally small-scale local investigations, of which the majority (71.0%) were from the urban area, and a considerable proportion (44.7%) were from coastal cities^20^. As indicated in this study and previous studies, hyperuricemia is generally more common in economically developed areas, for example, urban areas and coastal cities in China’s socioeconomic context^23,24^. In addition, a positive association of seafood consumption and hyperuricemia has been well-proven in coastal cities^25^. Given the fact that more than half of the Chines population were in rural areas and more than 90% of the Chinese cities are inland or remote cities, the results in that meta-analysis may overstate the prevalence of hyperuricemia in China, and a lower prevalence of hyperuricemia is expected for the whole nation^26,27^. By taking more less-developed areas into account, it is not surprising that the China National Survey of Chronic Kidney Disease, a survey covering 13 selected provinces, reported a lower prevalence of hyperuricemia than that in the systematic review and meta-analysis (8.4% vs. 13.3%), and similarly, our nationally representative prevalence of hyperuricemia was a bit lower than that in the China National Survey of Chronic Kidney Disease (6.4% vs. 8.4%)^21^.

In accordance with previous studies, age was confirmed as a significant risk factor for hyperuricemia in our study, the prevalence of hyperuricemia increased from the age of 60 years, and reached a plateau after the age of 70 years^4,11,21^. However, the effect of advanced age on hyperuricemia was slightly different between sexes, with older females at a higher risk of hyperuricemia than older males. This disparity may be largely related to the loss of the uricosuric action of oestrogen following the menopause^4^. In other words, the disparity of hyperuricemia prevalence between sexes seems to narrow with advanced age. But even so, hyperuricemia was still a male-dominated disease as indicated in our study.

Other socioeconomic factors were also found to be associated with the risk of hyperuricemia. Interestingly, the influence of marital status on hyperuricemia was exactly the opposite in males and females: single males were at a lower risk of hyperuricemia than married males, while single females were more likely to have hyperuricemia than married females. This discrepancy may arise from the difference in dietary intake and health behaviours that were associated with marital status. Given that females are generally in charge of food preparation and production in Chinese society, males’ dietary choices are more dependent on their sponsors compared to females^28,29^. Moreover, males and elderly remain a priority in domestic food distribution in Chinese circumstances, even when food supply is in short^28^. This phenomenon can also in part explain the positive association between economic improvement and hyperuricemia in females, which males were free from.

In this study, a regional diversity of hyperuricemia prevalence has been observed in both sexes, although demographic and socioeconomic features, and chronic conditions had all been accounted for. The variation might be a reflection of the regional food consumption patterns: the dominate dietary pattern is a carbohydrate-rich pattern in North China, and a “traditional Southern” (rice, vegetables, meat and poultry) pattern in Southern China^30^. The dietary effect of meat and poultry on elevated SUA has been discovered in previous studies, therefore it is plausible to observe this geographic diversity of hyperuricemia prevalence in our study^25^.

Some studies have revealed that smoking lowers the level of SUA, and this inverse association between smoking and SUA becomes obvious when the smoking duration exceeds 5 years^31,32^. In our study, smoking was detected as a risk factor for hyperuricemia only in females. This association in females could be explained by fewer pack-years among females compared to males. In a similar dose-response approach, drinking, a proven risk factor for hyperuricemia^33,34^, was only found be associated with hyperuricemia in males in our study.

Similar to the findings in previous studies, hyperuricemia is associated with several common cardiovascular risk factors, including obesity and dyslipidemia. However, diabetes and hypertension were found to be only significantly associated with hyperuricemia in females, and no such associations were found in males. Previous studies have demonstrated a stronger association between hyperuricemia and cardiovascular risk in females than in males^5,9,35^. Therefore, this sex-specific difference indicated in our study might be due to the sex-specific physiologic impact of hyperuricemia, or the difference in the severity of diabetes and hypertension between males and females in our study^5,36^. However, this phenomenon is not without controversy: the Framingham Heart Study revealed that elevated levels of SUA were with a higher incidence of type 2 diabetes in both sexes^37^, and one study from Shanghai showed that the association of higher SUA and incident diabetes was stronger in males than in females^38^. Further studies are still needed to confirm those sex-specific associations and to detail the underlying mechanisms.

Several limitations of the study also need to be considered. First, this study only examined the prevalence of hyperuricemia in middle-aged and older Chinese. Given that hyperuricemia is not only a concern for middle-aged and older individuals, our findings could only be applied to Chinese adults aged 45 years and above, rather than the whole general population^39,40^. Second, the SUA level in the CHARLS was only evaluated once, and the hyperuricemic status was thus not verified whether it was transient or persistent. Third, the prevalence of hyperuricemia could only be generated for the six geographic regions in China at best, our ability to estimate the prevalence of hyperuricemia at the provincial level was largely limited because the CHARLS data were not representative for each sampled province. Fourth, regarding the associated factors and comorbidities of hyperuricemia, no causal inferences can be made due to the cross-sectional study design. Finally, only a limited number of possible associated factors were included for analysis, the effects of some well-proven correlates of hyperuricemia, such as food consumption, diuretic use, were not able to be assessed because of the availability of information.

In conclusion, our nationwide, population-based study showed that the prevalence of hyperuricemia was 6.4% among Chinese people aged 45 years and above, which is lower than previously expected. However, given the large population size and rapid ageing trend in China, a higher prevalence of hyperuricemia may be expected in the foreseen future.

## Methods

### Study Design and study population

The CHARLS is an on-going national household-based survey conducted by the National School for Development (China Centre for Economic Research) at Peking University. The study was approved by the Ethical Review Committee of Peking University, and signed informed consent was provided by all participants at the time of participation. This study was performed in accordance with relevant guidelines and regulations. The detailed descriptions of study design and implement have been published previously, and are available at the study website (http://charls.pku.edu.cn/en)^41,42^.

The CHARLS 2011 (baseline) was conducted between June 2011 and March 2012. Briefly, eligible samples were drawn through a four-stage, stratified, cluster sampling procedure. First, all county-level units in Mainland China (except Tibet province, Hainan province and Ningxia Hui Autonomous Region) were stratified by region, urban/ rural setting and economic status (per capita statistics on the gross domestic product). By using a probability-proportional-to-size (PPS) sampling technique, 150 representative counties out of 28 provinces were randomly selected; Second, three primary sampling units (PSU, communities in urban areas or administrative villages in rural areas) were randomly chosen from each of the 150 selected counties; Third, all the dwellings within every selected PSU were outlined on Google Earth maps using the specifically designed “CHARLS-GIS” software, among which at least 24 were randomly selected in each of the 450 selected PSUs; Finally, if there were more than one member aged 45 years or above within one selected household, one such member was randomly selected; In the meanwhile, the spouse of the selected individual was also interviewed. Overall, the household response rate was 80.5%, and 17,708 individual participants in 10,257 households successfully completed at least one module of the survey^42^.

### Data collection

Information on demographics (i.e. age, sex, residence), socioeconomic status (i.e. educational attainment, marital status, income and expenditure), medical history and health-related behaviours (i.e. smoking, drinking) was collected by using a structured questionnaire in the household interview^41,42^. Then, 78.9% (13,978 individuals) of all the study participants provided anthropometric and physical-performance measurements. Body height was measured to the nearest 0.1 centimetres without shoes (Seca^TM^ 213 stadiometer), and weight to the nearest 0.1 kilograms without shoes and in light clothes (Omron^TM^ HN-286 scale). Body mass index (BMI) was calculated by dividing body weight (in kilograms) by squared height (in metres). Systolic blood pressure (SBP) and diastolic blood pressure (DBP) were recorded to the nearest mmHg for three times at 45-second intervals (Omron^TM^ HEM-7200 Monitor)^42^. In addition, 66.9% (11,847 individuals) of the study participants provided venous blood samples (8 ml each individual). A complete blood count was conducted within 1–2 hours after blood collection, while a specimen of whole blood was stored at 4 °C for later testing. The remainder was collected to obtain plasma and buffy coat, and then stored at −20 °C. All the blood samples were shipped to the Chinese Center for Disease Control in Beijing within 2 weeks and placed in a deep freezer at −80 °C until being assayed at the Youanmen Center for Clinical Laboratory of Capital Medical University^42,43^.

### Definitions of hyperuricemia and other factors

The primary definition of hyperuricemia in this study was a level of SUA ≥7.0 mg/dl (416.0 μmol/l) in males or ≥6.0 mg/dl (357.0 μmol/l) in females^11,21^. We also presented the prevalence of hyperuricemia by using two alternative definitions of hyperuricemia, namely, an SUA level ≥7.0 mg/dl (above the supersaturation point) and an SUA level ≥6.0 mg/dl (a widely accepted therapeutic target) regardless of sex^44–46^.

All subjects were categorized into four age groups: 45–49 years, 50–59 years, 60–69 years and ≥70 years. The levels of educational attainment were classified as illiterate, literate (having some informal education, being able to read or write), primary education, and middle school education and above. Marital status included married or cohabiting and single. The economic status was assessed by using per capita expenditures (PCE), a better welfare indicator to measure household resources than income in developing countries^47,48^. Since PCE was distributed asymmetrically, the natural logarithm of PCE – Ln(PCE) was taken and discretized into terciles. To take into the inequality of economic development between urban and rural areas, the classification of economic status was made for urban and rural areas separately. According to the place of residence, all subjects were categorized into six geographic regions, namely, East China (Anhui, Fujian, Jiangsu, Jiangxi, Shandong, Shanghai Municipality, Zhejiang), North China (Beijing, Hebei, Inner Mongolia Autonomous Region, Shanxi, Tianjin Municipality), Northeast China (Heilongjiang, Jilin, Liaoning), Northwest China (Gansu, Ningxia Hui Autonomous Region, Qinghai, Shaanxi, Xinjiang Uyghur Autonomous Region), South Central China (Guangdong, Guangxi Zhuang Autonomous Region, Hainan, Henan, Hubei, Hunan), Southwest China (Chongqing Municipality, Guizhou, Sichuan, Tibet, Yunnan)^26^ (see Supplementary Figure S2).

Subjects were categorized as never smoker, former smoker, and current smoker according to their smoking status, and never drinker, former drinker, and current drinker according to drinking status. Subjects were grouped by BMI as underweight (BMI < 18·5 kg/m^2^), normal (BMI from 18.5 to 22.9 kg/m^2^), overweight (BMI from 23.0 to 27.4 kg/m^2^) and obesity (BMI ≥ 27.5 kg/m^2^)^49^. Diabetes was defined as a self-reported physician-diagnosed diabetes, or a fasting blood glucose ≥ 126 mg/dl, or a random blood glucose ≥ 200 mg/dl, or an HbA_1c_ concentration of 6.5% or above, or currently taking antidiabetic medications^50^. Hypertension was defined as a self-reported physician-diagnosed hypertension, or an SBP ≥ 140 mmHg, or a DBP ≥ 90 mmHg, or currently being on antihypertensive medications^51^. Dyslipidemia was defined as a self-reported physician-diagnosed dyslipidemia, or an elevated TC level (≥6.2 mmol/l), or an elevated LDL cholesterol (≥4.1 mmol/l), or a low HDL cholesterol (<1.0 mmol/l), or an elevated TG (≥2.3 mmol/l), or taking antidyslipidemic medications^52^.

### Statistical analysis

To produce population representative estimates, we took into account the complex survey design and adopted the blood weight with household and individual response adjustment provided by the CHARLS^41,43^. All calculations in this study were weighted. The overall and gender-specific weighted values of SUA and prevalence of hyperuricemia were estimated for the overall population and for different subgroups according to strata of demographic characteristics (age), socioeconomic status (education, marriage, setting [urban and rural], economic status), and geographic region. In addition, the age-standardized values of SUA and prevalence of hyperuricemia were calculated by the direct method for the overall population, males and females respectively, after age standardisation to the Chinese census population in 2010^26^. Univariable and multivariable logistic regressions were used to examine the associations of demographic characteristics (age), socioeconomic status (education, marriage, setting [urban and rural], economic status), geographic location (geographic region), health behaviours (smoking, drinking), comorbidities (obesity, diabetes, hypertension and dyslipidemia) with the odds of hyperuricemia, for males and females respectively.

Continuous data were presented as means with standard deviations (SDs), and categorical data as proportions with 95% confidence intervals (CIs). All data analyses were performed using Stata statistical software (version 14.0; Stata Corporation, College Station, TX, USA). Maps were drawn using ArcMap version 10.1 (Environmental Systems Research Institute, Redlands, CA, USA), and the China base map was obtained as shapefile from the Global Administrative Area database (GADM, 2015, version 2.0; www.gadm.org). All statistical tests were 2-sided. A P value less than 0.05 was considered as statistically significant.

## Electronic supplementary material

Supplementary information
