# Supplementary material for: Prevalence and correlates of hyperuricemia in the middle-aged and older adults in China
Source: Sci Rep. 2018 Mar 12;8:4314. doi: 10.1038/s41598-018-22570-9 (PMC5847518; doi:10.1038/s41598-018-22570-9)
Supplement: Supplementary file 1 — Supplementary information [file 41598_2018_22570_MOESM1_ESM.docx]

Title: Prevalence and correlates of hyperuricemia in the middle-aged and older adults in China

Authors: Peige Song,^1,2^ He Wang,^3^ Wei Xia,^4^ Xinlei Chang,^1^ Manli Wang,^1^ Lin An^1,*^

^1^ Department of Maternal and Child Health, School of Public Health, Peking University, Beijing, China. ^2^ Centre for Population Health Sciences, University of Edinburgh, Edinburgh, UK ^3^ The First Affiliated Hospital of Zhengzhou University, Zhengzhou, China ^4^ School of Nursing, University of Hong Kong, Hong Kong, China. Correspondence and requests for materials should be addressed to L.A. (anlinbjmu@163.com).


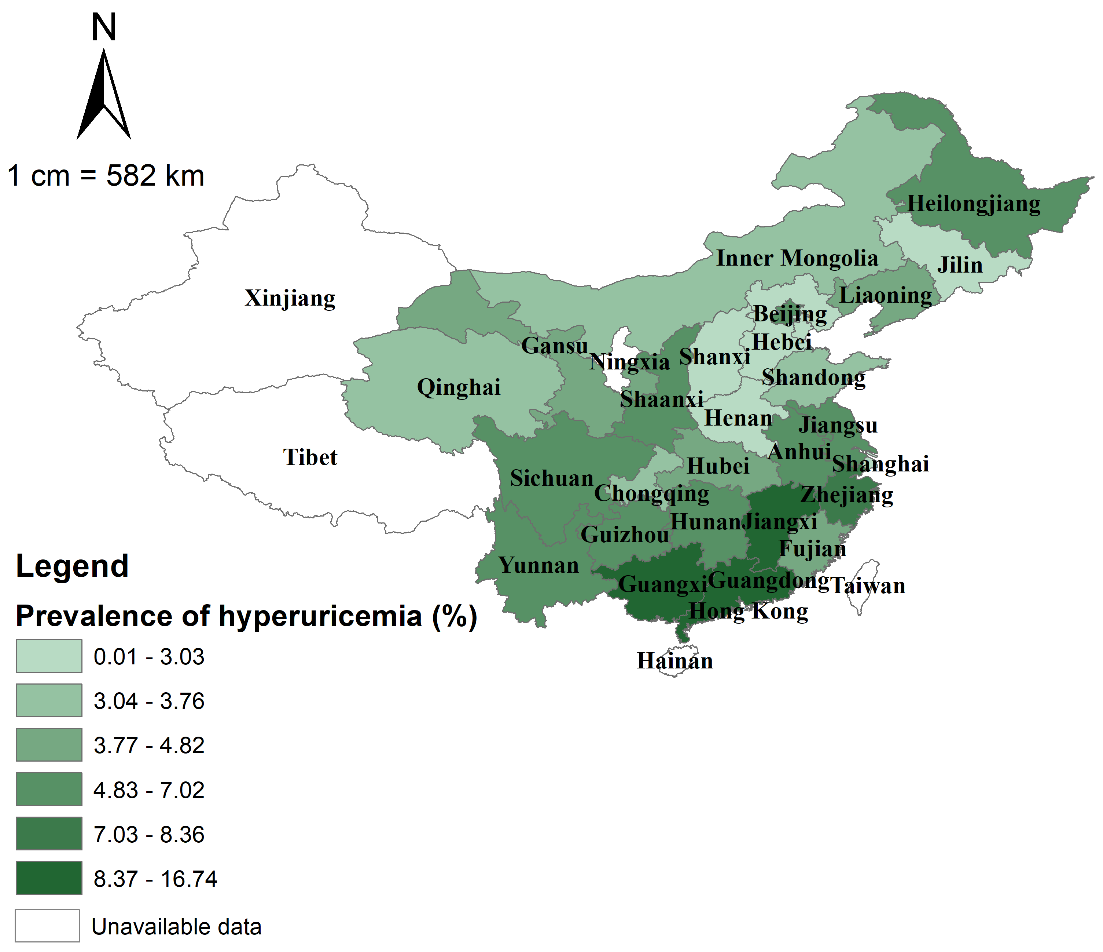


**Supplementary Figure S1**. Prevalence of hyperuricemia in the CHARLS 2011 survey, by province. The map was created using ArcMap (version 10.1, ESRI Inc. Redlands, CA, USA. https://www.esri.com/).


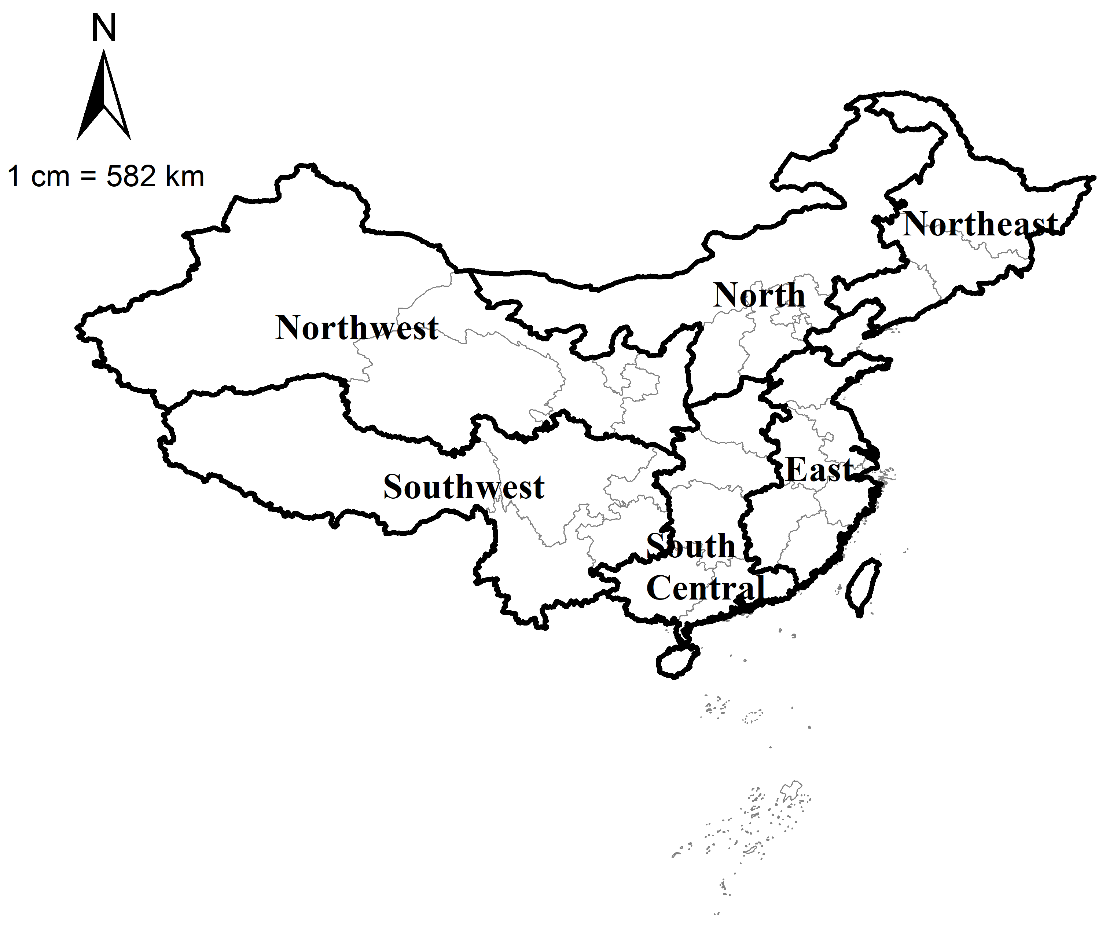


**Supplementary Figure S2**. The six geographic regions in China. The map was created using ArcMap (version 10.1, ESRI Inc. Redlands, CA, USA. https://www.esri.com/).

**Supplementary Table S1**. Comparison of general characteristics between the included and non-included subjects in the CHARLS 2011 survey*

| **Characteristic** | **Non-included subjects (n=2,067)** | **Included subjects (n=9,557)** | **Sample (n=11,624)** | **P value^‡^** |
| --- | --- | --- | --- | --- |
| **Age group** |  |  |  | 0.106 |
| 45-49 years | 19.7 | 21.9 | 21.4 |  |
| 50-59 years | 32.0 | 34.5 | 34.0 |  |
| 60-69 years | 25.5 | 25.5 | 25.5 |  |
| ≥70 years | 22.8 | 18.2 | 19.1 |  |
| **Sex** |  |  |  | 0.944 |
| Male | 48.6 | 48.8 | 48.8 |  |
| Female | 51.4 | 51.2 | 51.2 |  |
| **Education** |  |  |  | 0.353 |
| Illiterate | 26.4 | 24.9 | 25.2 |  |
| Literate | 15.5 | 17.1 | 16.8 |  |
| Primary education | 19.2 | 21.9 | 21.4 |  |
| Middle school education and above | 38.9 | 36.1 | 36.6 |  |
| **Marital status** |  |  |  | 0.004 |
| Married or cohabiting | 83.1 | 87.3 | 86.4 |  |
| Single | 16.9 | 12.7 | 13.6 |  |
| **Ln(PCE) by setting****^†^** |  |  |  | 0.778 |
| *Rural* |  |  |  |  |
| Bottom tertile | 24.2 | 18.1 | 18.2 |  |
| Middle tertile | 18.9 | 17.5 | 17.6 |  |
| Top tertile | 13.5 | 17.6 | 17.5 |  |
| *Urban* |  |  |  |  |
| Bottom tertile | 9.8 | 13.1 | 13.0 |  |
| Middle tertile | 17.9 | 14.5 | 14.6 |  |
| Top tertile | 15.7 | 19.2 | 19.1 |  |
| **Region** |  |  |  | 0.034 |
| East China | 35.6 | 28.9 | 30.2 |  |
| North China | 6.9 | 12.8 | 11.6 |  |
| Northeast China | 5.0 | 9.0 | 8.1 |  |
| Northwest China | 6.0 | 8.1 | 7.7 |  |
| South Central China | 32.1 | 25.2 | 26.6 |  |
| Southwest China | 14.4 | 16.1 | 15.7 |  |

^*^ Values were the weighted proportion. PCE=per capita expenditure.

^‡^ Comparison between included and non-included subjects.

^†^ The bottom tertile refers to the poor, the middle tertile refers to the middle, and the top tertile refers to the rich.

**Supplementary Table S2**. Prevalence of elevated serum uric acid among the included subjects in the CHARLS 2011 survey^*^

|  | **Prevalence of elevated serum uric acid (serum uric acid≥7mg/dl), % (95% CI)** | | | **Prevalence of elevated serum uric acid (serum uric acid≥6mg/dl), % (95% CI)** | | |
| --- | --- | --- | --- | --- | --- | --- |
|  | **Male** | **Female** | **Overall** | **Male** | **Female** | **Overall** |
| **Crude** | 7.9 (5.8-10.0) | 1.3 (0.8-1.8) | 4.5 (3.4-5.6) | 20.5 (17.9-23.2) | 4.9 (3.9-5.9) | 12.5 (11.1-14.0) |
| **Age-standardized** | 7.8 (5.8-9.8) | 1.2 (0.8-1.7) | 4.4 (3.4-5.5) | 20.4 (17.8-22.9) | 4.8 (3.8-5.7) | 12.4 (10.9-13.8) |
| **Age group** |  |  |  |  |  |  |
| 45-49 years | 4.8 (3.1-6.4) | 0.5 (0.0-0.9) | 2.5 (1.7-3.3) | 18.2 (13.6-22.8) | 2.4 (1.2-3.5) | 9.8 (7.6-11.9) |
| 50-59 years | 7.0 (4.1-9.9) | 0.7 (0.3-1.2) | 3.8 (2.3-5.3) | 16.7 (12.8-20.6) | 3.8 (2.5-5.0) | 10.2 (8.0-12.3) |
| 60-69 years | 7.9 (6.0-9.8) | 2.0 (0.9-3.0) | 4.9 (3.8-6.0) | 20.9 (17.4-24.3) | 6.8 (4.7-8.9) | 13.8 (11.6-16.0) |
| ≥70 years | 13.3 (4.1-22.4) | 2.4 (1.2-3.7) | 7.7 (2.9-12.5) | 30.3 (21.7-38.8) | 7.6 (5.2-10.0) | 18.6 (13.7-23.5) |
| **Education** |  |  |  |  |  |  |
| Illiterate | 5.9 (3.8-8.1) | 1.6 (0.8-2.4) | 2.6 (1.8-3.4) | 16.3 (12.4-20.3) | 5.2 (3.9-6.5) | 7.7 (6.2-9.2) |
| Literate | 6.8 (4.5-9.2) | 1.4 (0.5-2.3) | 4.2 (2.9-5.4) | 20.2 (15.6-24.9) | 5.1 (3.1-7.2) | 12.8 (10.2-15.4) |
| Primary education | 11.2 (4.1-18.4) | 1.3 (0.4-2.3) | 7.0 (2.9-11.1) | 23.9 (17.2-30.7) | 4.1 (2.1-6.0) | 15.4 (11.3-19.4) |
| Middle school education and above | 6.9 (5.1-8.8) | 0.7 (0.3-1.2) | 4.5 (3.3-5.7) | 19.8 (16.5-23.1) | 5.0 (3.2-6.7) | 14.1 (11.8-16.3) |
| **Marital status** |  |  |  |  |  |  |
| Married or cohabiting | 8.1 (5.9-10.4) | 1.0 (0.6-1.4) | 4.6 (3.5-5.8) | 20.9 (18.1-23.7) | 4.2 (3.2-5.3) | 12.7 (11.1-14.3) |
| Single | 5.6 (3.2-7.9) | 2.7 (1.3-4.1) | 3.7 (2.4-4.9) | 17.2 (12.3-22.1) | 8.4 (5.8-11.0) | 11.4 (9.0-13.7) |
| **Ln(PCE) by setting^†^** |  |  |  |  |  |  |
| *Rural* | *6.1 (4.9-7.3)* | *1.1 (0.7-1.6)* | *3.6 (2.9-4.2)* | *16.6 (14.2-19.0)* | *3.9 (2.9-4.9)* | *10.1 (8.7-11.6)* |
| Bottom tertile | 4.9 (3.3-6.4) | 0.8 (0.2-1.4) | 2.7 (2.0-3.5) | 14.2 (11.3-17.1) | 2.4 (1.47-3.3) | 8.0 (6.5-9.6) |
| Middle tertile | 6.3 (4.7-7.9) | 1.6 (0.7-2.5) | 3.9 (3.0-4.9) | 16.0 (12.8-19.2) | 4.7 (3.1-6.4) | 10.3 (8.3-12.4) |
| Top tertile | 7.1 (4.9-9.3) | 1.0 (0.3-1.7) | 4.1 (2.9-5.2) | 19.6 (16.2-23.0) | 4.6 (2.9-6.4) | 12.1 (10.1-14.0) |
| *Urban* | *10.0 (6.0-14.0)* | *1.5 (0.7-2.2)* | *5.6 (3.6-7.6)* | *25.1 (20.6-29.6)* | *6.0 (4.3-7.8)* | *15.3 (12.9-17.7)* |
| Bottom tertile | 8.2 (4.9-11.4) | 1.3 (0.0-2.5) | 4.5 (2.5-6.5) | 21.5 (16.8-26.1) | 5.9 (3.2-8.6) | 13.2 (10.2-16.1) |
| Middle tertile | 8.7 (4.0-13.3) | 2.3 (1.0-3.6) | 5.4 (2.9-7.9) | 27.1 (20.2-33.9) | 7.0 (4.6-9.4) | 16.8 (12.8-20.7) |
| Top tertile | 12.2 (4.7-19.7) | 0.9 (0.2-1.6) | 6.5 (3.1-9.9) | 26.0 (18.4-33.5) | 5.5 (2.7-8.2) | 15.6 (12.5-18.7) |
| **Region** |  |  |  |  |  |  |
| East China | 7.5 (5.2-9.8) | 1.4 (0.6-2.2) | 4.3 (3.3-5.4) | 19.9 (15.2-24.6) | 5.3 (3.4-7.2) | 12.3 (9.9-14.8) |
| North China | 3.5 (2.2-4.9) | 0.5 (0.0-1.0) | 2.0 (1.2-2.7) | 13.5 (8.9-18.2) | 2.8 (1.5-4.1) | 8.0 (5.6-10.5) |
| Northeast China | 5.0 (1.9-8.0) | 0.8 (0.0-1.8) | 2.8 (1.3-4.4) | 16.2 (10.5-21.8) | 4.5 (1.6-7.5) | 10.2 (6.5-13.9) |
| Northwest China | 8.0 (2.8-13.1) | 0.3 (0.0-0.8) | 4.2 (1.3-7.0) | 18.2 (8.1-28.4) | 2.4 (0.0-5.0) | 10.4 (4.9-16.0) |
| South Central China | 12.7 (6.0-19.4) | 1.8 (0.4-3.2) | 7.1 (3.6-10.5) | 26.0 (19.5-32.5) | 5.7 (3.0-8.4) | 15.5 (11.6-19.3) |
| Southwest China | 6.4 (4.9-7.9) | 1.6 (0.6-2.6) | 4.0 (3.2-4.8) | 22.5 (18.6-26.5) | 6.0 (4.8-7.3) | 14.3 (12.3-16.3) |

^*^ Values were weighted. 95% CI=95% confidence interval PCE=per capita expenditure.

^†^ The bottom tertile refers to the poor, the middle tertile refers to the middle, and the top tertile refers to the rich.
